# Metabolite Signatures in Postpartum Prediabetes and Diabetes Among Indian Women With Prior Gestational Diabetes Mellitus: Insights From CHIP‐F Study

**DOI:** 10.1002/dmrr.70232

**Published:** 2026-09-24

**Authors:** Sahil Kapoor, Ambika Singh, Neelam Mahlawat, J. Manivannan, Arpita Singh, Shinjini Bhatnagar, Nikhil Tandon, Yashdeep Gupta, Pallavi Kshetrapal

**Affiliations:** ^1^ Lab of Perinatal Research Department of Maternal and Child Health BRIC‐Translational Health Science and Technology Institute (BRIC‐THSTI) Faridabad Haryana India; ^2^ The Mount Sinai Hospital New York New York USA; ^3^ Department of Endocrinology & Metabolism All India Institute of Medical Sciences (AIIMS) New Delhi India

**Keywords:** biomarker, GDM, machine learning, metabolomics, pathophysiology, postpartum, T2DM

## Abstract

**Aims:**

Gestational diabetes mellitus (GDM) increases the risk of postpartum dysglycaemia. This study investigates candidate metabolites and perturbed metabolic pathways associated with postpartum prediabetes/diabetes among high‐risk Indian/South‐Asian women with prior GDM, a largely unexplored population.

**Materials and Methods:**

In this nested case‐control study within the Cohort of Indian Women with Hyperglycaemia in Pregnancy and their Families (CHIP‐F), untargeted metabolomics (UPLC‐MS/MS) was performed on plasma samples from 216 women with prior GDM, which included 22 participants with postpartum diabetes, 88 with prediabetes and 106 with normoglycaemia.

**Results:**

Untargeted metabolomics combined with machine learning approach revealed distinct metabolic alterations associated with postpartum dysglycaemia (prediabetes/diabetes) among women with prior GDM. After adjustment for age, body mass index, family history of diabetes, and postpartum duration, each 1‐standard deviation increase in the log‐transformed concentrations of Tyrosine (aOR = 1.71, 95% CI: 1.01–2.91) and L‐Kynurenine (aOR = 1.69, 1.02–2.80) was associated with higher odds of diabetes. Similarly, higher concentrations of 2‐Aminoisobutyric acid (aOR = 1.50, 1.09–2.06), Butyrylcarnitine (aOR = 1.99, 1.44–2.75), L‐Norleucine (aOR = 1.96, 1.41–2.72), and Propionylcarnitine (aOR = 1.94, 1.41–2.67) were associated with higher odds of prediabetes. Pathway analysis revealed potential dysregulation of aromatic amino acids (AAAs) metabolism in prediabetes. In diabetes, in addition to dysregulation of AAAs, potential lipid metabolic disturbances were also present.

**Conclusions:**

This study identified distinct candidate metabolic signatures associated with postpartum prediabetes and diabetes in Indian/South‐Asian women with a history of prior GDM, highlighting early disruptions in AAAs and lipid metabolism.

## Graphical abstract

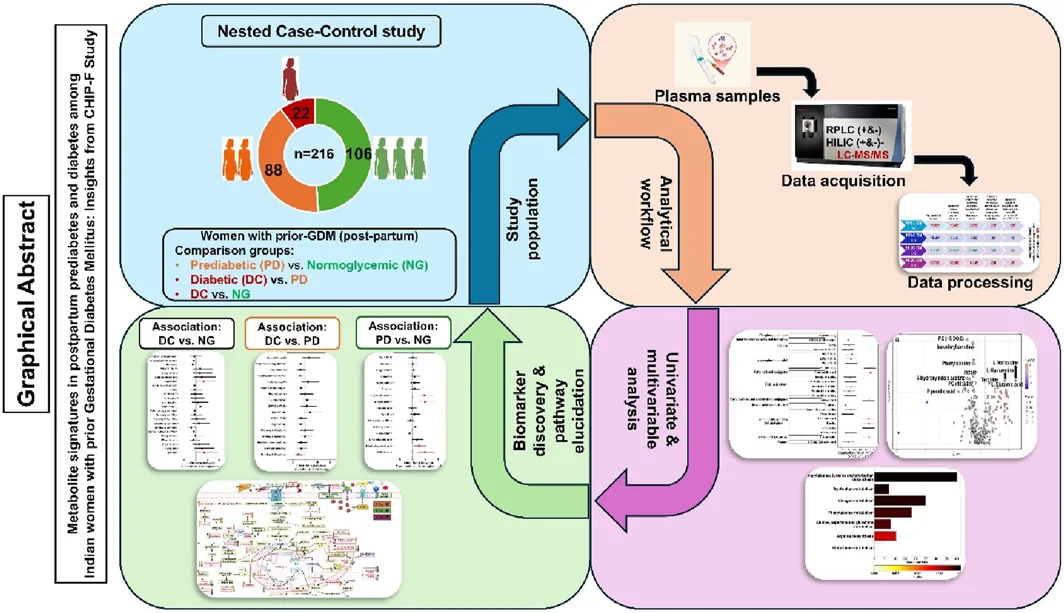

## 1 Introduction

GDM is one of the common metabolic complications during pregnancy, affecting approximately 1 in 7 pregnancies worldwide [1]. Women with GDM face a significantly higher risk (10–18 times greater) of developing postpartum diabetes compared with those normoglycemic during pregnancy [2]. This risk is particularly pronounced among South‐Asian women compared with other ethnic groups [3, 4, 5]. Furthermore, GDM is linked to a substantial burden of cardiometabolic risk factors, increasing the likelihood of cardiovascular diseases [6].

To mitigate the risk of type 2 diabetes and cardiovascular diseases, it is essential to undergo regular screenings. Clinical guidelines recommend using a 75‐g Oral Glucose Tolerance Test (OGTT) between 4 and 12 weeks postpartum, followed by annual screenings [7]. However, postpartum screening rates remain alarmingly low (10%) in routine care [8]. Barriers to the widespread adoption of OGTT include the test's lengthy duration and the requirement to consume 75‐g glucose [7]. Alternative tests, such as fasting plasma glucose or HbA1c, are available but have lower sensitivity in detecting early variance, potentially missing opportunities for timely intervention.

Given these challenges, there is an increasing interest in identifying surrogate biomarkers with diagnostic accuracy of the 75‐g OGTT, particularly for postpartum prediction. The risk of progressing to prediabetes or type 2 diabetes after GDM is heterogeneous, yet current tools such as OGTT and HbA1c classify women into broad categories (IFG, IGT, or normoglycaemia) that overlook the metabolic differences driving individual risk trajectories [9], highlighting the value of metabolic markers. This therefore underscores the urgent need for molecular signatures that more accurately reflect the underlying pathophysiology to enable personalised risk stratification and therapeutic interventions in mothers with prior GDM.

To address this crucial gap, the present study investigates the candidate metabolites associated with postpartum prediabetes and diabetes among women with prior GDM. Metabolomics has emerged as a powerful tool for identifying predictive signatures and elucidating disease mechanisms, including pathways involved in diabetes progression [10]. Previous research has highlighted strong associations between the transition from GDM to type 2 diabetes, identifying metabolic shifts such as increased fatty acid biosynthesis and amino acid metabolism [11, 12, 13]. However, the specific metabolites associated with postpartum dysglycaemia in Indian/South‐Asian women remain largely unexplored. Given that South‐Asian women exhibit heightened vulnerability to type 2 diabetes due to factors such as low lean mass, reduced insulin secretion capacity, and increased ectopic fat deposition [14, 15], the lack of data in this population underscores the need for further investigation. Despite recent studies that provide important insights, the biochemical pathways driving dysglycaemia post‐delivery in mothers with prior GDM remain insufficiently understood. Thus, we hypothesise that women with prior GDM who progress to postpartum dysglycaemia exhibit distinct metabolic signatures compared with those women who sustain or regain normoglycaemia. This study aims to reveal potential candidate metabolites and elucidate the potential metabolic mechanisms contributing to postpartum prediabetes and diabetes among South‐Asian women with prior GDM.

## 2 Materials and Methods

### 2.1 Study Design and Ethics

This nested case (prediabetes and diabetes)‐control (normoglycaemic) study within the Cohort of Indian Women with Hyperglycaemia in Pregnancy and their Families (CHIP‐F) cohort investigated women who were diagnosed with GDM during pregnancy [16]. Postpartum follow‐up and biospecimen collection were conducted between 2019 and 2022. Ethical approvals were obtained for the CHIP‐F cohort from the Ethics Committee, AIIMS, New Delhi (IEC‐79/01.02.2019, RP‐38/2019). The ethics approvals for the current objectives have been obtained from both participating institutions, AIIMS, New Delhi, and BRIC‐THSTI, Faridabad, for the Intramural grant Ref. No.: IEC‐540/02.08.2019.

### 2.2 Inclusion & Exclusion Criteria

Study participants included women who were diagnosed with GDM in their index pregnancy using the International Association of the Diabetes and Pregnancy Study Groups (IADPSG) criteria [17]. The postpartum period (enrolment in this study) was from 4 months to 9 years post‐childbirth. Women on pharmacotherapy for diabetes, or who had an underlying illness were excluded from the study.

This study was part of our established cohort (CHIP‐F), in which we enrolled 1019 participants with different degrees of glycaemia in pregnancy. Of the 1019 participants enrolled in the study, 643 were identified as women with GDM according to the IADPSG criteria. Of these, 27 participants receiving pharmacotherapy were excluded, leaving 616 participants for further evaluation. Subsequently, 57 participants with pre‐existing chronic illnesses were excluded, resulting in a cohort of 559 participants. Among these, 155 participants who had only one abnormal value of the three glycaemic assessments were further excluded. The reason for this was to have robust categories for prediabetes and diabetes, and to avoid potential overlap with the control group (normoglycemia with all three values being normal). Of the remaining 404 participants, 216 were randomly selected for the study. The sensitivity analysis was performed between included and excluded participants for key demographic, antenatal and postpartum variables, which include prespecified variables, and we found no significant difference between the two groups (Supporting Information S1: Table S1 and Figure S1).

### 2.3 Definition of Glycaemic Measures

Postpartum glycaemic status was defined as per the American Diabetes Association (ADA) [7]. For prediabetes, we used two abnormal tests rather than one, as mentioned in the previous paragraph (Section 2.2). Details on sample collection, processing, anthropometric measures, lipid parameters, and insulin indices have been described previously [5, 16].

### 2.4 Sample Size Calculation

A priori sample size was computed with a significance level (alpha) of 0.05 and statistical power of 0.80 using the G‐Power software v3.1 [18]. A large effect size (Cohen's *d* = 0.8) was used for sample size estimation for Diabetes (DC) versus Normoglycaemia (NG) and DC versus Prediabetes (PD) groups, and a medium effect size (Cohen's *d* = 0.5) for the PD versus NG group. The allocation ratio was determined based on the prevalence rate of prediabetes and diabetes, as reported in our previous study [16].

### 2.5 Metabolite Extraction

A total of 45 μL of plasma was mixed with 200 μL of a methanol:acetone:acetonitrile solution (1:1:1) spiked with 2.5 μM of L‐Phenyl‐d5‐alanine internal standard (Sigma‐Aldrich, St. Louis, MO, United States). After an overnight incubation at −20°C, the samples were centrifuged at 14,000 g to pellet the proteins. The supernatant was collected and evaporated under a stream of nitrogen gas. This lyophilised sample was reconstituted in 100 μL of 50% methanol solution (Sigma‐Aldrich, St. Louis, MO, United States) and was passed through column filter tubes (0.22 μm) to remove impurities. Finally, 40 μL of the filtered sample was analysed in duplicate using ultra‐performance liquid chromatography‐electrospray ionization‐mass spectrometry (UPLC‐ESI‐MS/MS).

### 2.6 UPLC‐ESI‐MS/MS‐Based Metabolite Profiling

Metabolite profiling was conducted using a Thermo Scientific Ultimate 3000 LC system coupled with an Orbitrap Fusion Tribrid Mass Spectrometer (Thermo Fisher Scientific Inc., Waltham, MA, United States). Metabolites were separated using both reverse‐phase liquid chromatography (RPLC (C18)) and hydrophilic interaction liquid chromatography (HILIC) column (Supporting Information S1: Table S2a).

Data acquisition was performed in both electrospray positive and negative ionization modes, with a scan range of 65–1000 m/z. MS/MS conditions are elaborated in Supporting Information S1: Table S2b.

### 2.7 Metabolomics Data Handling and Processing

The LC‐MS/MS data were processed using MS‐DIAL ver. 4.9.2 on 23 predefined parameters identified based on a prior literature review (Supporting Information S1: Table S3) [19, 20, 21]. Metabolite annotation was performed using the ALL‐GNPS (Global Natural Products Social Molecular Networking) reference spectral library. Candidate metabolites were annotated based on MS/MS spectral matching using an identification score > 75%, a precursor mass tolerance of 5 ppm, and a retention time tolerance of 0.01 min. All annotated metabolites were considered putatively identified (MSI Level 2) according to the Metabolomics Standards Initiative (MSI) guidelines (Supporting Information S1: Table S3) [19, 20, 21, 22, 23]. A pooled quality control (QC) sample was injected after every 10 study samples to monitor instrument stability and reproducibility. L‐Phenyl‐d5‐alanine (2.5 μM) was used as the internal standard to monitor analytical performance. Following feature extraction, signals detected in blanks were removed, and duplicate features were excluded. Batch effects and peak area drift were corrected using MetaboDrift version 1.1 [24]. Metabolites with a CV ≤ 30% were retained for subsequent normalisation [20]. Data from RPLC and HILIC ESI (+/) modes were merged, log‐transformed, and normalised by median across glycaemic groups (PD vs. NG, DC vs. NG, DC vs. PD) using Metaboanalyst 6.0 [25].

### 2.8 Statistical Analysis

#### 2.8.1 Univariate Analysis

Metaboanalyst was used to identify differentially expressed metabolites (DEMs) across glycaemic groups through univariate analysis. Significant metabolites were determined based on a fold‐change (FC) threshold of ≥ 1.2 and a false discovery rate (FDR) adjusted *p*‐value ≤ 0.05 (Supporting Information S1: Table S4). Volcano plots were used to visualise DEM distribution, and forest plots to highlight standardized mean differences (FDR ≤ 0.05) using Jupyter notebook 7.0.8 [26, 27]. The metabolites were classified using ClassyFire [28].

#### 2.8.2 Association Analysis

Metabolites that remained statistically significant after false discovery rate (FDR) correction in the univariate analysis were used for multivariable machine learning‐based models. Prior to modelling, metabolite intensities were log‐transformed and standardized to z‐scores. Logistic regression analyses were performed to examine the association of these metabolites with glycaemic status using Jupyter notebook 7.0.8 [26, 27, 29]. The models were adjusted for age, body mass index (BMI), family history of diabetes, and postpartum duration, to account for their potential confounding effects on the associations between metabolic profiles and glycaemic status. Associations are reported as unadjusted odds ratio (OR) and adjusted odds ratio (aOR) with 95% confidence intervals (CIs), and were visualised using forest plots. ORs were interpreted per one standard deviation increase in log‐transformed metabolite intensities, with OR > 1 indicating higher odds, OR < 1 indicating lower odds, and OR = 1 indicating no association with prediabetes or diabetes.

#### 2.8.3 Sensitivity Analysis

Sensitivity analysis was performed using Stata 15.0 (Stata Corp. LP, College Station, TX, United States).

### 2.9 Pathway and Enrichment Analysis

Pathway analysis was conducted using the significant metabolites identified through univariate analyses. The Kyoto Encyclopaedia of Genes and Genomes (KEGG) database on Metaboanalyst was utilised for this analysis. Topology measurement was performed using relative betweenness centrality, while the hypergeometric test was applied for enrichment analysis (Impact: 0.4–1, *p* ≤ 0.05). Scatter plot was employed to visualise the pathways based on significant features. Additionally, metabolite set enrichment analysis (MSEA) was carried out using the KEGG database to identify enriched pathways associated with significant metabolites (*p* ≤ 0.05) [25].

## 3 Results

### 3.1 Population Characteristics

Metabolite profiling was performed on 216 women diagnosed with prior GDM. Among them, 22 had diabetes, 88 had prediabetes, and 106 had normoglycaemia at the time of enrolment into the study in their postpartum period. The participants had a mean age (SD) of 33.6 (5.2) years and a BMI of 27.5 (5.3) kg/m^2^. A family history of diabetes was reported in 101 women (46.8%). Baseline characteristics were similar between women selected for this sub‐study and those excluded (Table 1; Supporting Information S1: Table S1).

**TABLE 1:** Baseline characteristics of study participants.

Variable	Participants (*n* = 216)
Age (years)	33.60 ± 5.2
Period of gestation at diagnosis (weeks)	22.10 ± 5.8
Postpartum interval (months)	32 (22–49)
Education, graduate or above	149 (69.0)
Working status, employed	46 (21.3)
Either insulin or metformin use during pregnancy	64 (29.8)
Family history of diabetes	101 (46.8)
Postpartum metabolic parameters	
Fasting plasma glucose (mmol/L)	5.70 ± 1.3
2‐hr OGTT plasma glucose (mmol/L)	7.20 ± 2.5
Body mass index (kg/m^2^)	27.50 ± 5.3
Systolic blood pressure (mmHg)	112.90 ± 10.6
Diastolic blood pressure (mmHg)	75.00 ± 8.0
Total cholesterol (mmol/L)	4.40 ± 0.95
Low‐density lipoprotein cholesterol (mmol/L)	2.45 ± 0.74
High‐density lipoprotein cholesterol (mmol/L)	1.36 ± 0.35
Triglycerides (mmol/L)	1.12 (0.83, 1.39)

*Note:* Data expressed as number (%), mean ± SD, median (IQR), as appropriate.

### 3.2 Comprehensive Metabolomic Profiling and Key Feature Identification

A total of 75,023, 71,687, 95,128, and 96,720 features were detected in RPLC ESI (+), RPLC ESI (−), HILIC ESI (+), and HILIC ESI (−) modes, respectively through spectral data pre‐processing. The data processing resulted in 320 MS/MS annotated metabolites (RPLC ESI (+): 135, RPLC ESI (−): 42, HILIC ESI (+): 44, HILIC ESI (−): 99) (Supporting Information S1: Figure S2). These metabolites were analysed using univariate and multivariable machine learning models to identify group‐specific differences and associations.

### 3.3 Univariate Analysis

**FIGURE 1:**
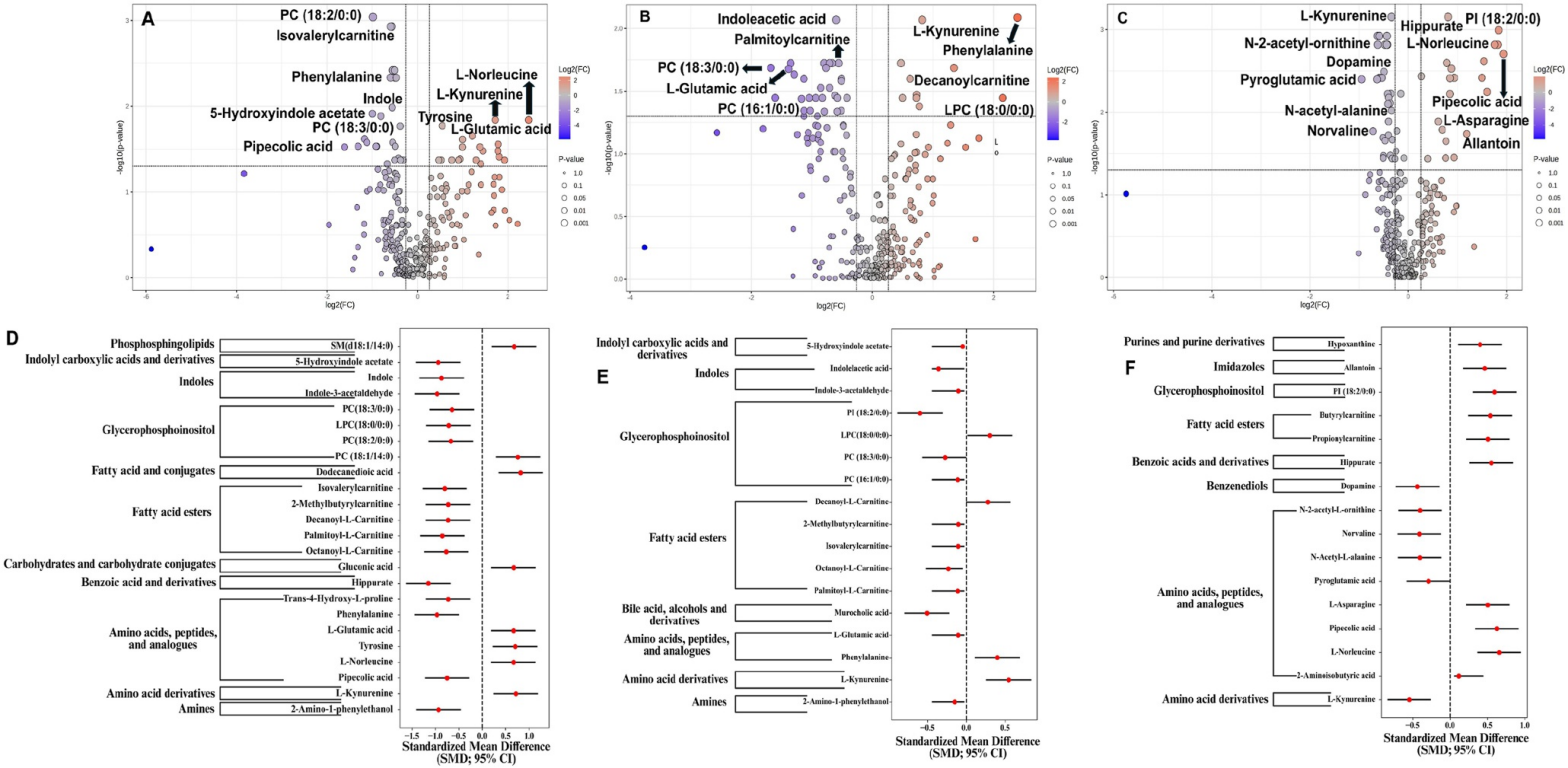
Volcano plot showing differentially expressed metabolites (DEMs) across glycaemic groups: (A) Diabetes versus Normoglycaemia (8 upregulated, 16 downregulated), (B) Diabetes versus Prediabetes (4 upregulated, 13 downregulated), and (C) Prediabetes versus Normoglycaemia (10 upregulated, 6 downregulated). Orange dots represent upregulated DEMs, while purple dots indicate downregulated DEMs. Forest plot illustrating the standardized mean difference (SMD) in normalised peak intensity of key metabolites across three glycaemic groups: (D) Diabetes versus Normoglycemic (E) Diabetes versus Prediabetes, and (F) Prediabetes versus Normoglycaemia. Metabolites with significant differences (FDR‐adjusted *p*‐value ≤ 0.05) are highlighted with red circles (Created using BioRender).

Univariate analysis identified 24 differentially expressed metabolites (DEMs) in DC versus NG (8 upregulated, 16 downregulated), 17 DEMs in DC versus PD (4 upregulated, 13 downregulated), and 16 DEMs in PD versus NG (10 upregulated, 6 downregulated) (FDR adjusted *p*‐value ≤ 0.05, Figure 1A–C, Supporting Information S1: Table S5). The forest plot revealed distinct metabolic changes associated with various glycaemic groups, including reduced levels of indoles and lipids, reported previously with insulin resistance and mitochondrial dysfunction. Phenylalanine was increased in the diabetes group compared with the prediabetes group, reflecting insulin resistance. L‐Kynurenine level was decreased in the prediabetes group, indicating potential inflammatory involvement (Figure 1D–F).

### 3.4 Association Analysis

#### 3.4.1 Diabetes Versus Normoglycaemia

**FIGURE 2:**
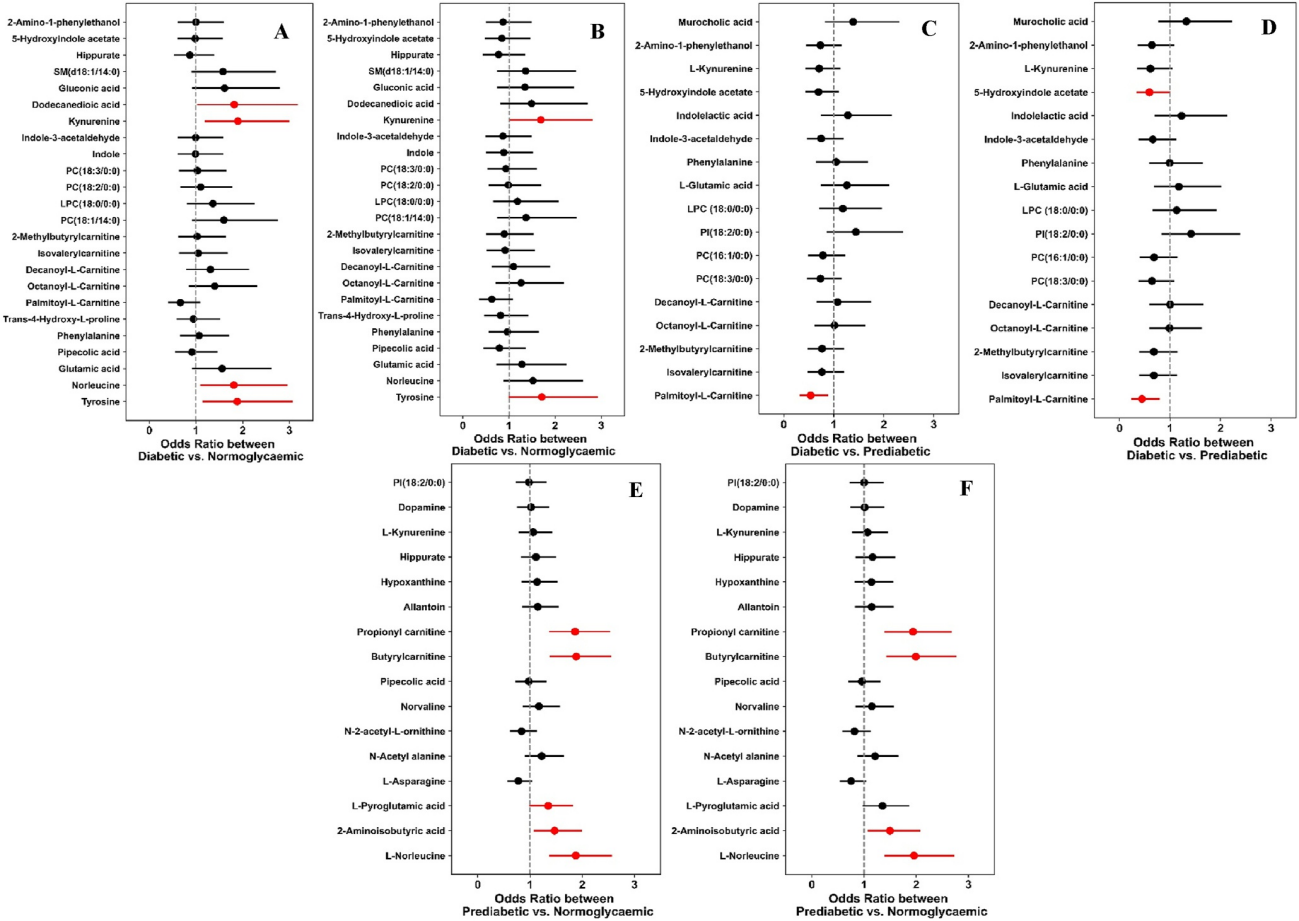
Forest plots illustrating the unadjusted and adjusted (adjusted for age, body mass index (BMI), family history of diabetes, and postpartum duration as covariaties) odds ratio at 95% confidence interval for the association between metabolite intensities and glycaemic status across the three glycaemic groups (Diabetes vs. Normoglycemia: (A‐B) Diabetes vs. Prediabetes: (C‐D) Prediabetes vs. Normoglycaemia: (E‐F) based on a logistic regression model. Panel (A, C, E) shows unadjusted odds ratio and Panel (B, D, F) shows adjusted odds ratio. The vertical dashed line indicates the null value (OR = 1). Red markers depict significant associations (*p* ≤ 0.05) and black markers indicate non‐significant associations (*p* > 0.05).

In the unadjusted and adjusted logistic regression analyses, higher levels of L‐Kynurenine and Tyrosine were significantly associated with increased odds of diabetes. For each 1‐standard deviation (SD) increase in the log‐transformed concentration of L‐Kynurenine, the odds of diabetes increased by 90% in the unadjusted model (OR = 1.90, 95% CI: 1.21–2.98, *p* = 0.01) and 69% in the adjusted model (aOR = 1.69, 95% CI: 1.02–2.80, *p* = 0.04) (Figure 2A–B, Supporting Information S1: Table S6a). Similarly, each 1‐SD increase in the log‐transformed concentration of Tyrosine was associated with 88% higher odds of diabetes in the unadjusted model (OR = 1.88, 95% CI: 1.16–3.06, *p* = 0.01) and 71% higher odds in the adjusted model (aOR = 1.71, 95% CI: 1.01–2.91, *p* = 0.05) (Figure 2A–B, Supporting Information S1: Table S6a).

However, Norleucine and Dodecanedioic acid were significantly associated with increased odds of diabetes only in the unadjusted analysis. Each 1‐SD increase in the log‐transformed metabolite concentration was associated with 81% higher odds of diabetes for Norleucine (OR = 1.81, 95% CI: 1.11–2.94, *p* = 0.02) and 82% higher odds for Dodecanedioic acid (OR = 1.82, 95% CI: 1.05–3.16, *p* = 0.03) (Figure 2A, Supporting Information S1: Table S6a).

#### 3.4.2 Diabetes Versus Prediabetes

In the unadjusted and adjusted logistic regression analyses, higher levels of Palmitoyl‐L‐carnitine were significantly associated with decreased odds of diabetes. For each 1‐SD increase in the log‐transformed metabolite concentration of Palmitoyl‐L‐carnitine, the odds of diabetes decreased by 46% in the unadjusted model (OR = 0.54, 95% CI: 0.33–0.86, *p* = 0.01) and 56% in the adjusted model (aOR = 0.44, 95% CI: 0.25–0.78, *p* = 0.00) (Figure 2C–D, Supporting Information S1: Table S6b).

Interestingly, 5‐Hydroxyindole acetate was significantly associated with decreased odds of diabetes only in the adjusted analysis. Each 1‐SD increase in the log‐transformed concentration of 5‐Hydroxyindole acetate was associated with 41% decreased odds of diabetes (aOR = 0.59, 95% CI: 0.35–0.99, *p* = 0.05) (Figure 2C, Supporting Information S1: Table S6b).

#### 3.4.3 Prediabetes Versus Normoglycaemia

Higher levels of L‐Norleucine, 2‐Aminoisobutyric acid, Butyryl carnitine and Propionyl carnitine were significantly associated with increased odds of prediabetes in both the unadjusted and adjusted logistic regression analyses. For each 1‐SD increase in the log‐transformed concentration of L‐Norleucine, the odds of prediabetes increased by 88% in the unadjusted model (OR = 1.88, 95% CI: 1.38–2.55, *p* = 0.00) and 96% in the adjusted model (aOR = 1.96, 95% CI: 1.41–2.72, *p* = 0.00) (Figure 2E–F, Supporting Information S1: Table S6c). Similarly, each 1‐SD increase in the log‐transformed concentration of 2‐Aminoisobutyric acid was associated with 47% higher odds of prediabetes in the unadjusted model (OR = 1.47, 95% CI: 1.09–1.97, *p* = 0.01) and 50% higher odds in the adjusted model (aOR = 1.50, 95% CI: 1.09–2.06, *p* = 0.01) For each 1‐SD increase in the log‐transformed concentration of Butyryl carnitine, the odds of prediabetes increased by 89% in the unadjusted model (OR = 1.89, 95% CI: 1.40–2.54, *p* = 0.00) and 99% in the adjusted model (aOR = 1.99, 95% CI: 1.44–2.75, *p* = 0.00) (Figure 2E–F, Supporting Information S1: Table S6c). Similarly, each 1‐SD increase in the log‐transformed concentration of Propionyl carnitine was associated with 86% higher odds of prediabetes in the unadjusted model (OR = 1.86, 95% CI: 1.38–2.52, *p* = 0.00) and 94% higher odds in the adjusted model (aOR = 1.94, 95% CI: 1.41–2.67, *p* = 0.00).

In contrast, Pyroglutamic acid was significantly associated with increased odds of prediabetes only in the unadjusted analysis. Each 1‐SD increase in the log‐transformed concentration of Pyroglutamic acid was associated with 35% higher odds of prediabetes (OR = 1.35, 95% CI: 1.01–1.80, *p* = 0.04) (Figure 2E, Supporting Information S1: Table S6c).

### 3.5 Pathway and Enrichment Analysis

**FIGURE 3:**
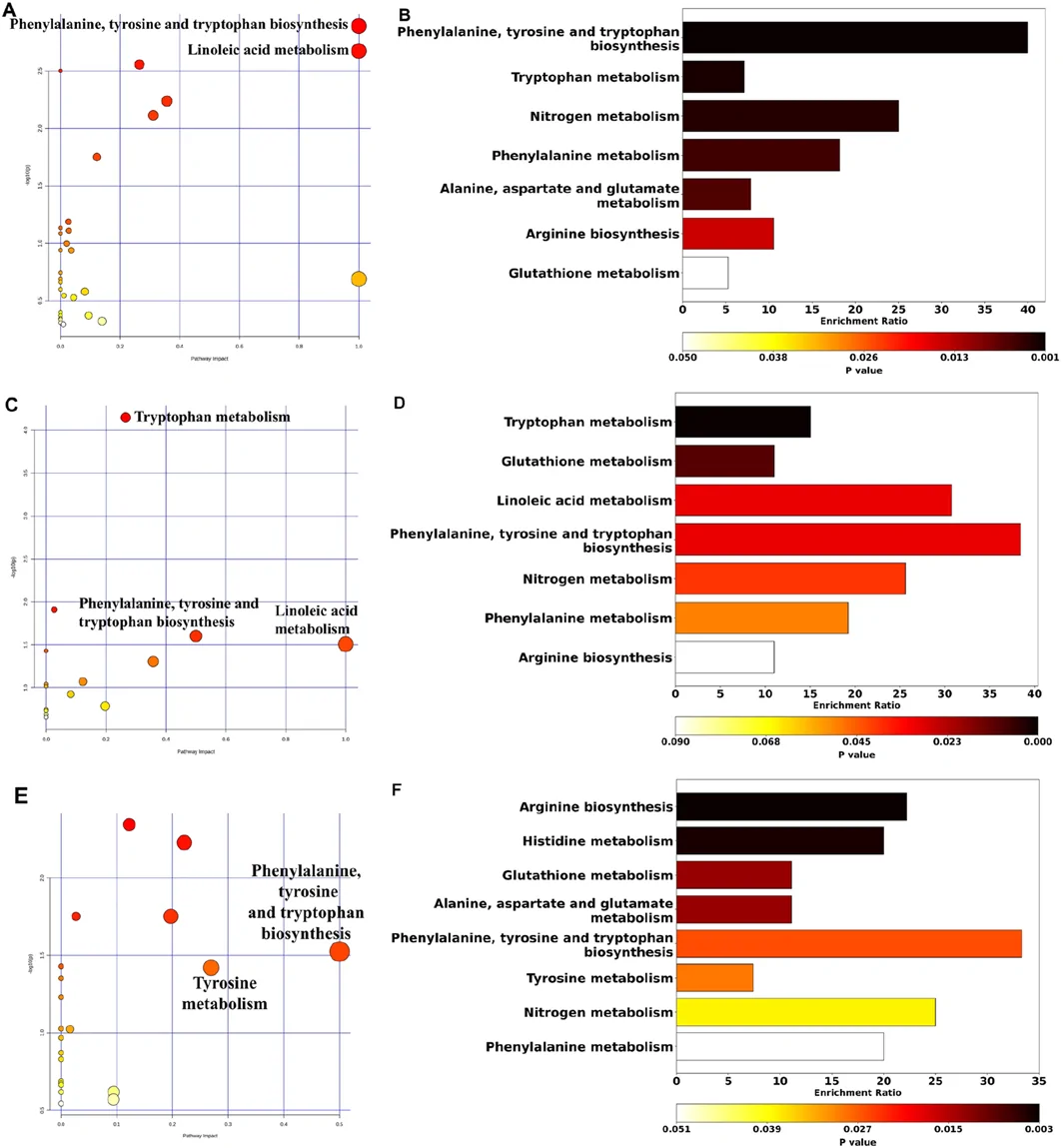
Pathway and enrichment analysis of significant DEMs across glycaemic groups: (A, B) Diabetes versus Normoglycaemia (C, D) Diabetes versus Prediabetes, and (E, F) Prediabetes versus Normoglycaemia (*p* ≤ 0.05).

Pathway and enrichment analyses of significant metabolites identified via univariate analysis across glycaemic groups (24 metabolites for DC vs. NG, 17 metabolites for DC vs. PD, and 16 metabolites for PD vs. NG) revealed notable dysregulation in metabolic pathways. In the diabetes group, dysregulation in both amino acid and lipid metabolism was highlighted, including pathways involving phenylalanine, tyrosine, tryptophan, and linoleic acid metabolism (impact: 0.6–1, *p* ≤ 0.05). In the prediabetes group, significant alterations were observed only in amino acid metabolism, including pathways involving phenylalanine, tyrosine, and tryptophan metabolism (impact: 0.5, *p* ≤ 0.05) (Figure 3A–F, Supporting Information S1: Table S7 and S8).

## 4 Discussion

**FIGURE 4:**
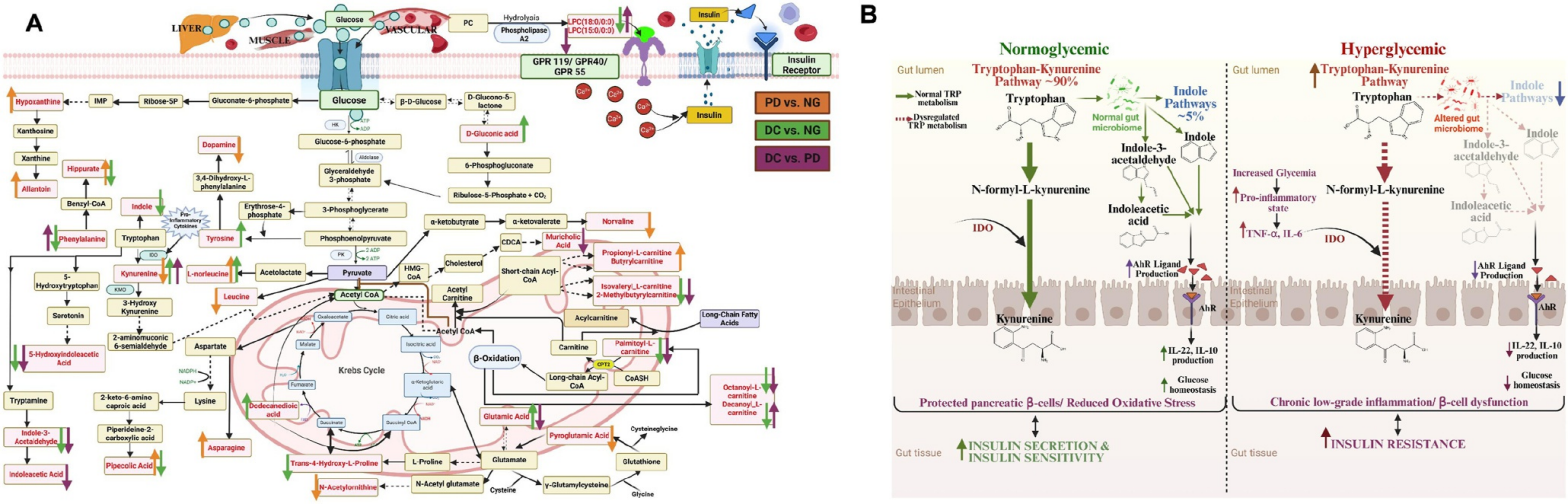
(A). Dysregulated biochemical pathways post‐GDM. Graphical representation of the hypothesis‐generating dysregulated metabolic pathways between the Prediabetes versus Normoglycaemia (orange arrow), Diabetes versus Normoglycaemia (green arrow), and Diabetes versus Prediabetes (purple arrow) groups from the post‐GDM cohort. Major pathways potentially altered during the transition from the normoglycemic state to type 2 diabetes are amino acid metabolism, tricarboxylic acid (TCA) cycle, fatty acid oxidation (FAO), lipid metabolism, and carnitine shuttle. Lysophosphatidylcholines (LPCs) are derived from phosphatidylcholine, which is formed mainly via Phospholipase A2 (PLA2) activity on phosphatidylcholine by Lecithin–Cholesterol acyltransferase (LCAT) in plasma. G‐protein‐coupled receptors GPR119, GPR40, and GPR55 are activated by LPCs, leading to the mobilisation and accumulation of intracellular cAMP. This affects insulin signalling and glucose absorption in adipose, muscle, and liver tissues, as well as controlling insulin release from the pancreatic β‐cells. The development of type 2 diabetes is influenced by dysregulation in LPC 18:0/0:0 and LPC 15:0/0:0, which are implicated in lipid metabolism, inflammation, insulin signalling, and mitochondrial function. In the carnitine shuttle, as Acylcarnitines are dysregulated, they may potentially disrupt insulin sensitivity, leading to an elevated ROS that causes insulin resistance and the development of diabetes (Created using BioRender). (B) Metabolic shift in the Tryptophan metabolism towards heightened Kynurenine pathway and dampened Indole pathway in the hyperglycaemic state. Tryptophan (TRP) is metabolised through three pathways: the Kynurenine (KYN) pathway (∼90%), the Indole pathway (5%), and the Serotonin pathway (2%). The TRP‐KYN pathway leads to the synthesis of KYN in the presence of Indoleamine 2,3‐dioxygenase (IDO) and its derivatives. Transition towards the glycaemic state induces pro‐inflammation, thereby increasing levels of TNF‐α and interleukin‐6 (IL‐6), which induces IDO activation. This leads to increased KYN levels in the plasma and the unavailability of TRP for the indole and serotonin pathways, resulting in a metabolic shift towards KYN, which contributes to insulin resistance (IR). In the indole pathway, TRP is catabolised by the gut microbiota into various metabolites, Indole, Indole‐3‐acetaldehyde, and Indoleacetic acid, that regulate glucose homeostasis via the AhR by upregulating IL‐22 and IL‐10, thus promoting insulin secretion and insulin sensitivity. Continuous green arrows indicate normal TRP metabolism, and dashed red arrows indicate dysregulated TRP metabolism (Created using BioRender).

Metabolic profiles offer valuable insights into the dysregulated physiological processes and underlying causes in the transition of a disease. At present, we have little understanding of the potential biomarkers that are associated with prediabetes or diabetes in South‐Asian women with prior GDM. To address this, we conducted a cross‐sectional untargeted metabolomics analysis on samples from women with a history of GDM. We identified 24 metabolites that were differentially expressed between the DC and NG groups, 17 DEMs in DC versus PD, and 16 DEMs in PD versus NG (Figure 4A).

A previous cross‐sectional study analysed women with prior GDM, in an ethnically diverse population primarily comprising White, Asian, and Hispanic individuals and identified 27 metabolites distinguishing type 2 diabetes from non‐type 2 diabetes using a targeted approach [11]. The study reported upregulation of metabolites such as glutamate and tyrosine, and downregulation of glycerophospholipids. Additionally, in a European cohort of women with prior GDM, NMR‐based profiling revealed higher levels of branched‐chain amino acids (BCAAs) and lower glycine concentrations in the type 2 diabetes group [30]. Lappas et al. [31], in a mixed population, identified three lipid species associated with type 2 diabetes development using targeted lipidomics.

Our study revealed distinct distribution patterns of metabolites across glycaemic groups, highlighting specific biochemical changes associated with different stages of the disease pathology. In the PD versus NG group, the amino acid‐related metabolites were found to be significantly dysregulated, suggesting early disturbances in amino acid metabolism, potentially correlating with the onset of insulin resistance. In diabetes, amino acids alongside fatty acid esters and other classes of metabolites were dysregulated, reflecting systemic remodelling in amino acid, lipid, and other metabolic pathways (Figure 4A). Lai et al. [11] also reported the metabolic dysregulation of amino acid and lipid metabolic networks, wherein they reported upregulation of amino acid metabolism, specifically BCAAs and arginine/proline metabolism, at baseline. While the major classes are consistent with previous findings, differences in the sub‐classes and in the identification of unique signatures highlight the ethnic variations among the study populations.

The current study findings indicate that key metabolic pathways involved in energy metabolism, including glycolysis, the tricarboxylic acid (TCA) cycle, fatty acid oxidation (FAO), and the carnitine shuttle, were dysregulated (Figure 4A), aligning with reports from previous global studies on post‐GDM and type 2 diabetes [11, 32, 33, 34, 35]. We found amino acids and derivatives such as Tyrosine, Kynurenine, and L‐Norleucine as well as lipids including Butyrylcarntine and Palmitoyl carnitine, to be primarily dysregulated across glycaemic groups.

Kynurenine (KYN), a key catabolic product of the Tryptophan‐Kynurenine (TRP‐KYN) metabolism pathway, plays a significant role in immune regulation, obesity, insulin resistance, and diabetes [36, 37]. Approximately 90% of TRP is converted into KYN via this pathway, while the remaining 5% enters the indole pathway or is synthesised into serotonin [38]. Current study findings display lower levels of KYN in the prediabetes group, whereas the diabetes group showed elevated levels. This pattern suggests that reduced KYN levels in the prediabetes stage may trigger a pro‐inflammatory response, potentially leading to impaired insulin signalling, beta‐cell dysfunction, and the onset of insulin resistance [39, 40]. The TRP‐KYN pathway in diabetes is a bidirectional feedback system that is influenced by the local concentration of secondary metabolites, cellular milieu, and the activity of enzymes. The pro‐inflammatory loop metabolites Quinolinic acid or 3‐Hydroxykynurenine reinforce inflammation and insulin resistance, while the anti‐inflammatory loop metabolites, Kynurenic acid and Picolinic acid, act as compensatory brakes and restore homeostasis [41, 42].

Conversely, the Indole pathway, which counterbalances the TRP‐KYN pathway, was found to be dysregulated in the diabetes group (Figure 4B). Specifically, metabolites such as Indole, Indole‐3‐acetaldehyde, and Indoleacetic acid were downregulated, as evident in the univariate analysis in the diabetes group, consistent with previous observations of reduced indole levels in type 2 diabetes [43]. Indole derivatives, known for their anti‐inflammatory properties, have shown therapeutic potential in reducing inflammation and blood glucose levels in high‐fat diet‐fed mice, making them promising targets for type 2 diabetes treatment [44].

Additionally, lower levels of metabolites such as, LPC (18:0/0:0) and PC (18:3/0:0) were observed in the diabetes group, aligning with earlier reports. These metabolites are associated with inflammation, oxidative stress, and dyslipidemia, which contribute to insulin resistance and beta‐cell dysfunction [45]. The observed reduction in long‐chain acylcarnitine and palmitoyl‐L‐carnitine levels in the diabetes group suggests impaired L‐carnitine availability, a metabolic disturbance commonly associated with uncontrolled diabetes. This deficiency compromises mitochondrial fatty acid transport and energy production, promoting metabolic dysregulation, insulin resistance, and the progression of chronic diabetic complications, including neuropathy [46, 47]. Conversely, elevated propionylcarnitine levels in the prediabetes group may serve as an early potential metabolic candidate of mitochondrial dysfunction and perturbed BCAAs catabolism. Such alterations are indicative of impaired oxidative metabolism and have been consistently associated with insulin resistance and an increased risk of cardiometabolic disease [48]. Consistent with previous findings, this study observed elevated levels of tyrosine in the diabetes group. Elevated levels of circulating AAAs have been associated with impaired fasting glucose and type 2 diabetes [49]. Elevated 2‐aminoisobutyric acid (AIB) levels observed in the prediabetes group may indicate an adaptive metabolic response during the early stages of dysglycaemia. Although direct evidence for AIB is limited, its increase may reflect pathways related to metabolic compensation, analogous to those described for β‐aminoisobutyric acid (BAIBA), which has been shown to improve insulin sensitivity, stimulate lipid oxidation, and suppress inflammation [50]. Furthermore, although L‐Norleucine has been identified as a biomarker of prediabetes [51], the role of this metabolite in the development of the post‐GDM to type 2 diabetes has not been clearly elucidated yet. Here, we report L‐norleucine as a novel metabolite associated with prediabetes in women with prior‐GDM.

Previous studies used targeted platforms to identify metabolite biomarkers; however, our study, with its agonistic, untargeted approach, offers novel insights into metabolic dysregulation and uncovers previously unreported metabolites across glycaemic groups in the Indian ethnic population.

## 5 Conclusion

In conclusion, this single‐centre untargeted metabolomics study provides an understanding of metabolite alterations among South Asian women with prior‐GDM and current prediabetes/diabetes, and potential dysregulations in pathophysiological pathways. This study identified key metabolites that were associated with higher odds of postpartum prediabetes and diabetes. It also identified dysregulation in amino acid and lipid metabolism associated with hyperglycaemia among Indian women with prior‐GDM. Despite certain limitations, such as the need for external validation generalised to other populations/regions and reliance on annotated metabolites, our study highlights stage‐specific exploratory metabolic signatures that distinguish prediabetes from diabetes. Further, these findings provide a foundation for future multisite longitudinal studies to develop and validate predictive models for the prediction of future diseases and targeted interventions.

## Author Contributions

**Sahil Kapoor:** writing – review and editing, data curation, formal analysis, methodology, software, visualization, validation, writing – original draft. **Ambika Singh:** writing – review and editing, data curation, formal analysis, methodology, software, visualization, validation, writing – original draft. **Neelam Mahlawat:** writing – review and editing, data curation, formal analysis, methodology, software, visualization, validation, writing – original draft. **J. Manivannan:** methodology, software. **Arpita Singh:** methodology, software, visualization. **Shinjini Bhatnagar:** conceptualization, project administration, resources, writing – review and editing, data curation, formal analysis. **Nikhil Tandon:** conceptualization, project administration, resources, writing – review and editing, data curation, formal analysis. **Yashdeep Gupta:** conceptualization, project administration, resources, writing – review and editing, data curation, formal analysis, methodology, funding acquisition, investigation, supervision. **Pallavi Kshetrapal:** conceptualization, project administration, resources, writing – review and editing, data curation, formal analysis, methodology, funding acquisition, investigation, supervision.

## Funding

The CHIP‐F cohort and sample collection were supported by the Indian Council of Medical Research (ICMR) under Grant 55/4/8/CARE‐YD/2018‐NCD‐II, with Dr. Nikhil Tandon as the primary recipient of this grant. The spectral data acquisition was supported through the intramural grant. Manpower support for data analysis was provided through funding from the Department of Biotechnology (DBT), Ministry of Science and Technology, Government of India, under Grant BT/PR49993/CMD/150/134/2023.

## Ethics Statement

Ethical approvals were obtained for the CHIP‐F cohort from the Ethics Committee, AIIMS, New Delhi (IEC‐79/01.02.2019, RP‐38/2019). The ethics approvals for the current objectives have been obtained from both participating institutions, AIIMS, New Delhi, and BRIC‐THSTI, Faridabad, for the Intramural grant Ref. No.: IEC‐540/02.08.2019.

## Conflicts of Interest

The authors declare no conflicts of interest.

## Supporting information


Supporting Information S1

## Data Availability

The data and resources underlying this article are available from the corresponding author on reasonable request.
