# Supplementary material for: Metabolite Signatures in Postpartum Prediabetes and Diabetes Among Indian Women With Prior Gestational Diabetes Mellitus: Insights From CHIP‐F Study
Source: Diabetes Metab Res Rev. 2026 Sep 24;42(6):e70232. doi: 10.1002/dmrr.70232 (PMC13602894; doi:10.1002/dmrr.70232)
Supplement: Supplementary file 1 [file DMRR-42-e70232-s001.docx]

**216** Participants (22 participants with postpartum diabetes, 88 with prediabetes and 106 with normoglycaemia according to ADA criteria)

**1019** Participants registered in CHIP-F (parent cohort) (Excluded (preexisting diabetes [PED] [n = 101], ODiP [n = 92], GDM [n = 643]), and normoglycemia (n = 183) in pregnancy

**643** Participants diagnosed with GDM at 26-28 weeks of gestation according to IADPSG criteria

**Exclusion: 27** Participants with diabetes and on pharmacotherapy

**616** Participants

**Exclusion: 57** Participants with chronic illness

**559** Participants

**Exclusion: 155** Participants with only one of the FPG/OGTT/HbA1C values deranged

**404** Participants

Randomly selected participants (Excluded 188 (no differences)

**Figure S1.** Flow diagram of participant recruitment, eligibility assessment, and final study population.

**Figure S2.** Workflow illustrating the processing of UPLC-MS/MS data utilizing MS-Dial and Metabodrift.

**Table S1.** Sensitivity analysis for nested case-control study.

| Variable | Included  (n=216) | Excluded  (n=188) | p-value |
| --- | --- | --- | --- |
| Age (years) | 33.6 ± 5.2 | 34.5 ± 4.9 | 0.08 |
| Period of gestation at diagnosis (weeks) | 22.1 ± 5.8 | 21.5 ± 6.1 | 0.41 |
| Postpartum interval (months) | 32 (22-49) | 32 (20-47) | 0.63 |
| Education, graduate or above | 149 (69.0) | 119 (63.6) | 0.26 |
| Working status, employed | 46 (21.3) | 51 (27.1) | 0.17 |
| Either of insulin or metformin during pregnancy | 64 (29.8) | 64 (34.0) | 0.36 |
| Family history of diabetes | 101 (46.8) | 93 (49.5) | 0.59 |
|  |  |  |  |
| Postpartum metabolic parameters: |  |  |  |
| Fasting plasma glucose (mmol/L) | 5.7 ± 1.3 | 5.9 ± 1.5 | 0.23 |
| 2-hr OGTT plasma glucose (mmol/L) | 7.2 ± 2.5 | 7.7 ± 3.3 | 0.11 |
| Body mass index (kg/m^2^) | 27.5 ± 5.3 | 28.3 ± 5.8 | 0.13 |
| Systolic blood pressure (mmHg) | 112.9 ± 10.6 | 113.8 ± 13.0 | 0.45 |
| Diastolic blood pressure (mmHg) | 75.0 ± 8.0 | 76.0 ± 10.1 | 0.28 |
| Total cholesterol (mmol/L) | 4.40 ± 0.95 | 4.43 ± 0.83 | 0.67 |
| Low-density lipoprotein cholesterol (mmol/L) | 2.45 ± 0.74 | 2.48 ± 0.71 | 0.71 |
| High-density lipoprotein cholesterol (mmol/L) | 1.36 ± 0.35 | 1.33 ± 0.33 | 0.49 |
| Triglycerides (mmol/L) | 1.12  (0.83, 1.39) | 1.19  (0.89, 1.61) | 0.10 |

Data expressed as number (%), mean ± SD, median (IQR), as appropriate

**Table S2a.** Liquid chromatography (LC) conditions used for analysis of extracted plasma samples.

| **RPLC Column details** | **Reverse phase column (Waters Acquity UPLC HSS T3 RPLC Column (100 Å, 2.1 mm x 100 mm))** | |
| --- | --- | --- |
| **Gradient run time (min)** | **Solvent A% (Water** **acid)** | **Solvent B % (Methanol with 0.1%**   **Formic acid)** |
| 0 | 99 | 1 |
| 1 | 85 | 15 |
| 4 | 65 | 35 |
| 7 | 5 | 95 |
| 10 | 99 | 1 |
| 14 | 99 | 1 |
| **HILIC Column details** | **Waters XBridge BEH Amide HILIC Column (2.5 µm, 2.1 mm x 100 mm))** | |
| **Gradient run time (min)** | **Solvent A % (20 mM ammonium acetate)** | **Solvent B % (100%)** |
| 0 | 15 | 85 |
| 3 | 70 | 30 |
| 12 | 98 | 2 |
| 15 | 98 | 2 |
| 16 | 15 | 85 |
| 23 | 15 | 85 |

**Table S2b.** Tandem mass spectrometry (MS/MS) conditions used for analysis of extracted plasma samples.

| Flow rate | 0.3 ml/min |
| --- | --- |
| Injection volume | 5 µl |
| Column temperature | 40°C |
| Capillary temperature | 300°C |
| Vaporizer temperature | 310°C |
| Spray voltage (Positive mode) | 3500 V |
| Spray voltage (Negative mode) | 3000 V |
| Sheath gas | 42 arbs |
| Auxiliary gas | 11 arbs |
| Resolution (MS) | 1,20,000 |
| Resolution (MS/MS) | 30,000 |

**Table S3.** Parameters used for processing of LC-MS/MS data of extracted plasma samples of post-GDM mothers using open-source software MS-DIAL ver. 4.9.2.

| **S.no** | **Parameter** | **Set value** |
| --- | --- | --- |
| **1** | **Ionization type** | Soft Ionization |
| **2** | **Separation type** | Chromatography |
| **3** | **MS method type** | Conventional |
| **4** | **Date type (MS1)** | Profile |
| **5** | **Data type (MS2)** | Profile |
| **6** | **Ion mode** | Positive ion mode and Negative ion mode |
| **7** | **Target omics** | Metabolomics |
| **8** | **MS1 tolerance (da)** | 0.0005 |
| **9** | **MS2 tolerance (da)** | 0.0005 |
| **10** | **MS1 mass range begin (da)** | 65 |
| **11** | **MS1 mass range end (da)** | 1000 |
| **12** | **MS/MS mass range begin (da)** | 65 |
| **13** | **MS/MS mass range end (da)** | 1000 |
| **14** | **Minimum peak height** | 10000 |
| **15** | **Mass slice width (da)** | 0.1 |
| **16** | **Smoothing method** | Linear weighted moving average |
| **17** | **Smoothing level** | 3 |
| **18** | **Minimum peak width** | 5 |
| **19** | **Sigma window value** | 0.5 |
| **20** | **MSP file input (reference database)** | ALL_GNPS (Identification score ≥ 75  %, mass tolerance ≥ 5ppm, retention   time tolerance = 0.01 min) |
| **21** | **Retention time tolerance (min)** | 0.01 |
| **22** | **Identification score cut off (%)** | 75 |
| **23** | **Selection of possible adducts** | M + H+, M + H- |

**Table S4.** FDR adjusted p-value, FC and ROC-AUC cut off limit used in univariate analysis in previous studies.

| **ROC-AUC** | **ROC-AUC (p-value)** | **FC** | **FDR (p-value)** | **Research paper** | **Reference** |
| --- | --- | --- | --- | --- | --- |
|  |  | 2 | 0.05 | Maternal obesity characterized by gestational diabetes increases the susceptibility of rat offspring to hepatic steatosis via a disrupted liver metabolome | [https://doi.org/10.1113/JP27042](https://doi.org/10.1113/JP270429) [9](https://doi.org/10.1113/JP270429) |
| > 0.8 | 0.05 | 2 | 0.05 | The integrated bioinformatic analysis identifies immune microenvironment- related potential biomarkers for patients with gestational diabetes mellitus | [https://doi.org/10.3389/fimmu.2](https://doi.org/10.3389/fimmu.2024.1296855) [024.1296855](https://doi.org/10.3389/fimmu.2024.1296855) |
| > 0.8 | 0.05 |  |  | Plasma lipidomics in early pregnancy and risk of gestational diabetes mellitus: a prospective nested case– control study in Chinese women | [https://doi.org/10.1093/ajcn/nqa](https://doi.org/10.1093/ajcn/nqab242) [b242](https://doi.org/10.1093/ajcn/nqab242) |
| > 0.8 | 0.05 |  |  | Exploration of predictive metabolic factors for gestational diabetes mellitus in Japanese women using metabolomic analysis | <https://doi.org/10.1111/jdi.12887> |

|  |  | 1.2 | 0.05 | Untargeted metabolomics of saliva in pregnant women with and without gestational diabetes mellitus and healthy non-pregnant women | [https://doi.org/10.3389/fcimb.20](https://doi.org/10.3389/fcimb.2023.1206462) [23.1206462](https://doi.org/10.3389/fcimb.2023.1206462) |
| --- | --- | --- | --- | --- | --- |
| > 0.77 | 0.05 |  |  | Identification of differential metabolites using untargeted metabolomics between gestational diabetes and normal pregnant women | [https://doi.org/10.1002/ijgo.1425](https://doi.org/10.1002/ijgo.14253) [3](https://doi.org/10.1002/ijgo.14253) |
|  |  | 1.5 | 0.05 | Obesity Connected Metabolic Changes in Type 2 Diabetic Patients Treated with Metformin | [https://doi.org/10.3389/fphar.20](https://doi.org/10.3389/fphar.2020.616157) [20.616157](https://doi.org/10.3389/fphar.2020.616157) |
|  |  |  |  | Urinary Metabolites Altered during the Third Trimester in Pregnancies Complicated by Gestational Diabetes Mellitus: Relationship with Potential Upcoming Metabolic Disorders | [https://doi.org/10.3390/ijms2005](https://doi.org/10.3390/ijms20051186) [1186](https://doi.org/10.3390/ijms20051186) |
|  |  | 1 | 0.05 | Discovery of metabolic biomarkers for gestational diabetes mellitus in a Chinese population | <https://doi.org/10.1186/s12986-021-00606-8> |
|  |  | 1.2 | 0.05 | Large-Scale Longitudinal Metabolomics Study Reveals Different Trimester-Specific Alterations of Metabolites in Relation to Gestational Diabetes Mellitus | [https://doi.org/10.1021/acs.jprot](https://doi.org/10.1021/acs.jproteome.8b00602) [eome.8b00602](https://doi.org/10.1021/acs.jproteome.8b00602) |
| > 0.7 | 0.05 |  |  | Comprehensive analysis of serum metabolites in gestational diabetes mellitus by UPLC/Q-TOF-MS | <https://doi.org/10.1007/s00216-015-9211-3> |
|  |  | 1.5 | 0.05 | Steroid profiling and genetic variants in Chinese women with gestational diabetes mellitus | [https://doi.org/10.1016/j.jsbmb.2](https://doi.org/10.1016/j.jsbmb.2021.105999) [021.105999](https://doi.org/10.1016/j.jsbmb.2021.105999) |
|  |  | 1.2 | 0.05 | Potential biomarkers identified in plasma of patients with gestational diabetes mellitus | <https://doi.org/10.1007/s11306-021-01851-x> |
|  |  | 1.5 | 0.05 | Serum Proteomic Signatures in Umbilical Cord Blood of Preterm Neonates Delivered by Women with Gestational Diabetes | [https://doi.org/10.2147/DMSO.S](https://doi.org/10.2147/DMSO.S406297) [406297](https://doi.org/10.2147/DMSO.S406297) |
|  |  | 1.2 | 0.05 | Serum metabolomics study of pregnant women with gestational diabetes mellitus based on LC-MS | [https://doi.org/10.1016/j.sjbs.20](https://doi.org/10.1016/j.sjbs.2019.09.016) [19.09.016](https://doi.org/10.1016/j.sjbs.2019.09.016) |
| > 0.7 | 0.05 | 1 | 0.05 | Metabolomic approaches to dissect dysregulated metabolism in the progression of pre-diabetes to T2DM | [https://doi.org/10.1039/d3mo00](https://doi.org/10.1039/d3mo00130j) [130j](https://doi.org/10.1039/d3mo00130j) |

**Table S5.** List of differentially expressed metabolites identified across different glycaemic groups using univariate analysis. The table reports

log(2) fold change (log(2)FC), false discovery rate–adjusted q-value, and area under the receiver operating characteristic curve (ROC-AUC) for each metabolite. Positive log(2)FC values indicate upregulation, whereas negative values indicate downregulation. Metabolites with q-values < 0.05 were considered statistically significant.

| **S.no** | **Metabolites** | **Direction of Comparison:** | | | **Direction of Comparison:** | | | **Direction of Comparison:** | | |
| --- | --- | --- | --- | --- | --- | --- | --- | --- | --- | --- |
|  |  | **Diabetic vs. Normoglycemic** | | | **Diabetic vs. Prediabetic** | | | **Prediabetic vs. Normoglycemic** | | |
|  |  | **Regulation** | **log2 (FC)** | **q- value** | **Regulation** | **log2 (FC)** | **q- value** | **Regulation** | **log2 (FC)** | **q- value** |
| **1** | Hippurate | **↓** | -1 | 0.001 | - | - | - | **↑** | 1.84 | 0.001 |
| **2** | PI (18:2/0:0) | - | - | - | **↓** | -0.8 | 0.021 | **↑** | 1.76 | 0.002 |
| **3** | 2- Aminoisobutyric acid | - | - | - | - | - | - | **↑** | 1.61 | 0.006 |
| **4** | L-Norleucine | **↑** | 1.92 | 0.042 | - | - | - | **↑** | 1.54 | 0.002 |
| **5** | Pipecolic acid | **↓** | -1.1 | 0.026 | - | - | - | **↑** | 0.93 | 0.004 |
| **6** | Butyrylcarnitine | - | - | - | - | - | - | **↑** | 0.93 | 0.035 |
| **7** | L-Asparagine | - | - | - | - | - | - | **↑** | 0.85 | 0.006 |
| **8** | Hypoxanthine | - | - | - | - | - | - | **↑** | 0.76 | 0.036 |
| **9** | Propionylcarnitin e | - | - | - | - | - | - | **↑** | 0.61 | 0.013 |
| **10** | Allantoin | - | - | - | - | - | - | **↑** | 0.26 | 0.004 |
| **11** | L-Kynurenine | **↑** | 1.38 | 0.03 | **↑** | 0.8 | 0.042 | **↓** | -0.3 | 0.036 |
| **12** | N-2-acetyl-L- ornithine | - | - | - | - | - | - | **↓** | -0.3 | 0.022 |
| **13** | Dopamine | - | - | - | - | - | - | **↓** | -0.4 | 0.013 |
| **14** | Pyroglutamic acid | - | - | - | - | - | - | **↓** | -0.4 | 0.006 |
| **15** | N-Acetyl-L- alanine | - | - | - | - | - | - | **↓** | -0.4 | 0.002 |
| **16** | Norvaline | - | - | - | - | - | - | **↓** | -0.5 | 0.003 |
| **17** | Tyrosine | **↑** | 1.78 | 0.033 | - | - | - | - | - | - |
| **18** | Dodecanedioic acid | **↑** | 1.72 | 0.014 | - | - | - | - | - | - |
| **19** | L-Glutamic acid | **↑** | 1.3 | 0.042 | **↓** | -0.6 | 0.034 | - | - | - |
| **20** | PC (18:1/14:0) | **↑** | 1 | 0.024 | - | - | - | - | - | - |
| **21** | SM(d18:1/14:0) | **↑** | 0.94 | 0.041 | - | - | - | - | - | - |
| **22** | Gluconic acid | **↑** | 0.91 | 0.042 | - | - | - | - | - | - |
| **23** | PC (18:2/0:0) | **↓** | -0.4 | 0.042 | - | - | - | - | - | - |
| **24** | Isovalerylcarnitin e | **↓** | -0.4 | 0.017 | **↓** | -1.7 | 0.021 | - | - | - |
| **25** | 2- Methylbutyrylcar nitine | **↓** | -0.5 | 0.029 | **↓** | -1.2 | 0.036 | - | - | - |
| **26** | 2-Amino-1- phenylethanol | **↓** | -0.5 | 0.005 | **↓** | -0.5 | 0.045 | - | - | - |
| **27** | Phenylalanine | **↓** | -0.5 | 0.004 | **↑** | 0.5 | 0.036 | - | - | - |
| **28** | Indole-3- acetaldehyde | **↓** | -0.6 | 0.004 | **↓** | -0.8 | 0.019 | - | - | - |
| **29** | Indole | **↓** | -0.6 | 0.01 | - | - | - | - | - | - |
| **30** | 5-Hydroxyindole acetate | **↓** | -0.6 | 0.005 | **↓** | -0.6 | 0.036 | - | - | - |
| **31** | PC (18:3/0:0) | **↓** | -0.7 | 0.047 | **↓** | -0.6 | 0.046 | - | - | - |
| **32** | Trans-4-Hydroxy-  L-proline | **↓** | -0.9 | 0.029 | - | - | - | - | - | - |
| **33** | Palmitoyl-L-  Carnitine | **↓** | -1 | 0.012 | **↓** | -0.6 | 0.019 | - | - | - |
| **34** | Octanoyl-L-  Carnitine | **↓** | -1.2 | 0.024 | **↓** | -1.1 | 0.025 | - | - | - |
| **35** | Decanoyl-L-  Carnitine | **↓** | -1.3 | 0.029 | **↑** | 0.7 | 0.036 | - | - | - |
| **36** | LPC (18:0/0:0) | **↓** | -1.6 | 0.03 | **↑** | 0.7 | 0.034 | - | - | - |
| **37** | Indolelactic acid | - | - | - | **↓** | -0.4 | 0.036 | - | - | - |
| **38** | Murocholic acid | - | - | - | **↓** | -0.5 | 0.026 | - | - | - |
| **39** | PC (16:1/0:0) | - | - | - | **↓** | -1.4 | 0.019 | - | - | - |

**Table S6a.** The unadjusted and adjusted odds ratio (OR) at 95% confidence interval (CI) for the association between metabolite intensities and glycaemic status (Diabetes vs. Normoglycemia) estimated using logistic regression model. Adjusted models include age, body mass index (BMI), family history of diabetes, and postpartum duration as covariates.

| **S.no** | **Metabolite** | **Class** | **Unadjusted Odds ratio** | | **Adjusted Odds ratio** | |
| --- | --- | --- | --- | --- | --- | --- |
|  |  |  | **OR (95% CI)** | **p-value** | **aOR (95% CI)** | **p-value** |
| 1 | **Tyrosine** | Amino acid and analogues | **1.881 (1.157, 3.058)** | **0.01** | **1.711 (1.006, 2.910)** | **0.05** |
| 2 | **Norleucine** |  | **1.811 (1.114, 2.943)** | **0.02** | 1.519 (0.890, 2.590) | 0.13 |
| 3 | Glutamic acid |  | 1.557 (0.932, 2.602) | 0.09 | 1.281 (0.738, 2.224) | 0.38 |
| 4 | Pipecolic acid |  | 0.913 (0.576, 1.449) | 0.70 | 0.792 (0.466, 1.347) | 0.39 |
| 5 | Phenylalanine |  | 1.068 (0.675, 1.690) | 0.78 | 0.965 (0.573, 1.625) | 0.89 |
| 6 | Trans-4-hydroxy-L-proline |  | 0.946 (0.598, 1.498) | 0.81 | 0.817 (0.474, 1.409) | 0.47 |
| 7 | Palmitoyl-L-carnitine | Fatty acid esters | 0.666 (0.415, 1.068) | 0.09 | 0.628 (0.371, 1.064) | 0.08 |
| 8 | Octanoyl-L-carnitine |  | 1.402 (0.855, 2.297) | 0.18 | 1.261 (0.731, 2.175) | 0.40 |
| 9 | Decanoyl-L-carnitine |  | 1.308 (0.808, 2.118) | 0.27 | 1.098 (0.644, 1.873) | 0.73 |
| 10 | Isovaleryl carnitine |  | 1.049 (0.661, 1.666) | 0.84 | 0.914 (0.539, 1.549) | 0.74 |
| 11 | 2-Methylbutyrylcarnitine |  | 1.023 (0.646, 1.622) | 0.92 | 0.896 (0.528, 1.521) | 0.68 |
| 12 | PC (18:1/14:0) | Glycerophosphoinositol | 1.597 (0.931, 2.740) | 0.09 | 1.368 (0.764, 2.450) | 0.29 |
| 13 | LPC (18:0/0:0) |  | 1.361 (0.827, 2.241) | 0.23 | 1.183 (0.681, 2.055) | 0.55 |
| 14 | PC (18:2/0:0) |  | 1.100 (0.689, 1.757) | 0.69 | 0.987 (0.577, 1.688) | 0.96 |
| 15 | PC (18:3/0:0) |  | 1.030 (0.649, 1.634) | 0.90 | 0.932 (0.547, 1.590) | 0.80 |
| 16 | Indole | Indoles | 0.989 (0.625, 1.564) | 0.96 | 0.885 (0.520, 1.506) | 0.65 |
| 17 | Indole-3-acetaldehyde |  | 0.993 (0.627, 1.571) | 0.98 | 0.866 (0.510, 1.473) | 0.60 |
| 18 | **Kynurenine** | Amino acid derivatives | **1.896 (1.208, 2.976)** | **0.01** | **1.692 (1.023, 2.797)** | **0.04** |
| 19 | **Dodecanedioic acid** | Fatty acid and conjugates | **1.818 (1.045, 3.161)** | **0.03** | 1.491 (0.829, 2.683) | 0.18 |
| 20 | Gluconic acid | Carbohydrate conjugates | 1.612 (0.936, 2.776) | 0.09 | 1.347 (0.757, 2.397) | 0.31 |
| 21 | SM (d18:1/14:0) | Phosphosphingolipids | 1.579 (0.923, 2.699) | 0.10 | 1.361 (0.762, 2.429) | 0.30 |
| 22 | Hippurate | Benzoic acid derivatives | 0.866 (0.548, 1.369) | 0.54 | 0.774 (0.450, 1.330) | 0.35 |
| 23 | 5-Hydroxyindole acetate | Indolyl carboxylic acids and derivatives | 0.983 (0.622, 1.555) | 0.94 | 0.846 (0.497, 1.439) | 0.54 |
| 24 | 2-Amino-1-phenylethanol | Amines | 0.999 (0.631, 1.582) | 1.00 | 0.871 (0.513, 1.479) | 0.61 |

**Table S6b.** The unadjusted and adjusted OR at 95% CI for the association between metabolite intensities and glycaemic status (Diabetes vs. Prediabetes) estimated using logistic regression model. Adjusted models include age, body mass index (BMI), family history of diabetes, and postpartum duration as covariates.

| **S.no** | **Metabolite** | **Class** | **Unadjusted Odds ratio** | | **Adjusted Odds ratio** | |
| --- | --- | --- | --- | --- | --- | --- |
|  |  |  | **OR (95% CI)** | **p-value** | **aOR (95% CI)** | **p-value** |
| 1 | **Palmitoyl-L-carnitine** | Fatty acid esters | **0.536 (0.333, 0.863)** | **0.01** | **0.443 (0.252, 0.780)** | **0.00** |
| 2 | Isovaleryl carnitine |  | 0.764 (0.490, 1.191) | 0.23 | 0.677 (0.408, 1.123) | 0.13 |
| 3 | 2-Methylbutyryl carnitine |  | 0.765 (0.491, 1.193) | 0.24 | 0.678 (0.408, 1.126) | 0.13 |
| 4 | Octanoyl-L-carnitine |  | 1.012 (0.633, 1.616) | 0.96 | 0.985 (0.603, 1.609) | 0.95 |
| 5 | Decanoyl-L-carnitine |  | 1.072 (0.667, 1.723) | 0.77 | 1.000 (0.609, 1.643) | 1.00 |
| 6 | PC (18:3/0:0) | Glycerophosphoinositol | 0.735 (0.474, 1.142) | 0.17 | 0.641 (0.385, 1.067) | 0.09 |
| 7 | PC (16:1/0:0) |  | 0.781 (0.503, 1.213) | 0.27 | 0.682 (0.414, 1.125) | 0.13 |
| 8 | PI (18:2/0:0) |  | 1.441 (0.876, 2.370) | 0.15 | 1.415 (0.844, 2.370) | 0.19 |
| 9 | LPC (18:0/0:0) |  | 1.181 (0.718, 1.942) | 0.51 | 1.129 (0.667, 1.912) | 0.65 |
| 10 | L-Glutamic acid | Amino acid and analogues | 1.261 (0.763, 2.085) | 0.37 | 1.173 (0.691, 1.990) | 0.55 |
| 11 | Phenylalanine |  | 1.047 (0.657, 1.667) | 0.85 | 0.988 (0.601, 1.626) | 0.96 |
| 12 | Indole-3-acetaldehyde | Indoles | 0.749 (0.478, 1.173) | 0.21 | 0.659 (0.392, 1.108) | 0.12 |
| 13 | Indolelacetic acid |  | 1.282 (0.766, 2.145) | 0.34 | 1.225 (0.712, 2.108) | 0.46 |
| 14 | **5-Hydroxyindole acetate** | Indolyl carboxylic acids and derivatives | 0.695 (0.446, 1.081) | 0.11 | **0.589 (0.350, 0.992)** | **0.05** |
| 15 | L-Kynurenine | Amino acid derivatives | 0.708 (0.453, 1.108) | 0.13 | 0.610 (0.360, 1.033) | 0.07 |
| 16 | 2-Amino-1-phenylethanol | Amines | 0.732 (0.469, 1.142) | 0.17 | 0.639 (0.380, 1.073) | 0.09 |
| 17 | Murocholic acid | Bile acid derivatives | 1.389 (0.841, 2.293) | 0.20 | 1.322 (0.789, 2.215) | 0.29 |

**Table S6c.** The unadjusted and adjusted OR at 95% CI for the association between metabolite intensities and glycaemic status (Prediabetes vs. Normoglycaemia) estimated using logistic regression model. Adjusted models include age, body mass index (BMI), family history of diabetes, and postpartum duration as covariates.

| **S.no** | **Metabolite** | **Class** | **Unadjusted Odds ratio** | | **Adjusted Odds ratio** | |
| --- | --- | --- | --- | --- | --- | --- |
|  |  |  | **OR (95% CI)** | **p-value** | **aOR (95% CI)** | **p-value** |
| 1 | **L-Norleucine** | Amino acid and analogues | **1.876 (1.382, 2.548)** | **0.00** | **1.958 (1.411, 2.717)** | **0.00** |
| 2 | **2-Aminoisobutyric acid** |  | **1.469 (1.093, 1.974)** | **0.01** | **1.496 (1.088, 2.058)** | **0.01** |
| 3 | **L-Pyroglutamic acid** |  | **1.348 (1.007, 1.804)** | **0.04** | 1.356 (0.992, 1.854) | 0.06 |
| 4 | L-Asparagine |  | 0.775 (0.582, 1.032) | 0.08 | 0.754 (0.556, 1.021) | 0.07 |
| 5 | N-Acetyl alanine |  | 1.223 (0.919, 1.628) | 0.17 | 1.212 (0.895, 1.641) | 0.21 |
| 6 | N-2-acetyl-L-ornithine |  | 0.839 (0.631, 1.116) | 0.23 | 0.816 (0.602, 1.107) | 0.19 |
| 7 | Norvaline |  | 1.171 (0.881, 1.556) | 0.28 | 1.150 (0.850, 1.556) | 0.37 |
| 8 | Pipecolic acid |  | 0.978 (0.737, 1.297) | 0.88 | 0.959 (0.710, 1.295) | 0.79 |
| 9 | **Butyryl carnitine** | Fatty acid esters | **1.885 (1.396, 2.544)** | **0.00** | **1.991 (1.443, 2.748)** | **0.00** |
| 10 | **Propionyl carnitine** |  | **1.863 (1.380, 2.515)** | **0.00** | **1.939 (1.410, 2.668)** | **0.00** |
| 11 | Allantoin | Imidazoles | 1.150 (0.865, 1.528) | 0.34 | 1.145 (0.846, 1.550) | 0.38 |
| 12 | Hypoxanthine | Purine derivatives | 1.136 (0.855, 1.510) | 0.38 | 1.142 (0.843, 1.547) | 0.39 |
| 13 | Hippurate | Benzoic acid derivatives | 1.114 (0.839, 1.480) | 0.45 | 1.163 (0.856, 1.580) | 0.33 |
| 14 | L-Kynurenine | Amino acid derivatives | 1.062 (0.800, 1.410) | 0.68 | 1.064 (0.786, 1.441) | 0.69 |
| 15 | Dopamine | Benzenediols | 1.020 (0.769, 1.353) | 0.89 | 1.012 (0.749, 1.369) | 0.94 |
| 16 | PI (18:2/0:0) | Glycerophosphoinositol | 0.981 (0.740, 1.302) | 0.90 | 1.003 (0.741, 1.356) | 0.99 |

**Table S7.** Pathway analysis of significant DEMs identified in Diabetic vs. Normoglycemic, Diabetic vs. Prediabetic and Prediabetic vs. Normoglycemic group using the KEGG database, relative betweenness centrality and hypergeometric test on Metaboanalyst 6.0.

| Metabolic pathways | Diabetic vs. Normoglycemic | | | | | | Diabetic vs. Prediabetic | | | | | | Prediabetic vs. Normoglycemic | | | | | |
| --- | --- | --- | --- | --- | --- | --- | --- | --- | --- | --- | --- | --- | --- | --- | --- | --- | --- | --- |
|  | **Total** | **Hits** | **Raw p** | **Holm adjust** | **FDR** | **Impact** | **Total** | **Hits** | **Raw p** | **Holm adjust** | **FDR** | **Impact** | **Total** | **Hits** | **Raw p** | **Holm adjust** | **FDR** | **Impact** |
| Phenylalanine, tyrosine and tryptophan biosynthesis | 4 | 2 | 0 | 0.1 | 0.06 | 1 | 4 | 1 | 0.02 | 1 | 0.59 | 0.5 | 4 | 1 | 0.03 | 1 | 0.43 | 0.5 |
| Arginine biosynthesis | 14 | 2 | 0.02 | 1 | 0.2 | 0.12 | 14 | 1 | 0.08 | 1 | 0.86 | 0.12 | 14 | 2 | 0 | 0.36 | 0.24 | 0.12 |
| Alanine, aspartate and glutamate metabolism | 28 | 3 | 0.01 | 0.58 | 0.1 | 0.31 | 28 | 1 | 0.16 | 1 | 1.00 | 0.20 | 28 | 2 | 0.02 | 1 | 0.35 | 0.2 |
| Linoleic acid metabolism | 5 | 2 | 0 | 0.17 | 0.06 | 1 | 5 | 1 | 0.03 | 1 | 0.59 | 1 |  |  |  |  |  |  |
| Phenylalanine metabolism | 8 | 2 | 0.01 | 0.44 | 0.09 | 0.36 | 8 | 1 | 0.05 | 1 | 0.66 | 0.36 |  |  |  |  |  |  |
| Tryptophan metabolism | 41 | 4 | 0 | 0.22 | 0.06 | 0.27 | 41 | 4 | 0.00 | 0.01 | 0.01 | 0.27 |  |  |  |  |  |  |
| Tyrosine metabolism | 42 | 1 | 0.48 | 1 | 1 | 0.14 |  |  |  |  |  |  | 42 | 2 | 0.04 | 1 | 0.43 | 0.27 |
| D-Amino acid metabolism | 15 | 1 | 0.2 | 1 | 0.96 | 1 |  |  |  |  |  |  |  |  |  |  |  |  |
| Histidine metabolism |  |  |  |  |  |  |  |  |  |  |  |  | 16 | 2 | 0.01 | 0.47 | 0.24 | 0.22 |

**Table S8.** Enrichment analysis of significant DEMs identified in Diabetic vs. Normoglycemic, Diabetic vs. Prediabetic and Prediabetic vs. Normoglycemic group using the KEGG database, relative betweenness centrality and hypergeometric test on Metaboanalyst 6.0 (p ≤ 0.05).

| **Metabolic pathway** | **Diabetes vs. Normoglycaemia** | | | | | | **Diabetes vs. Prediabetes** | | | | | | **Prediabetes vs. Normoglycaemia** | | | | | |
| --- | --- | --- | --- | --- | --- | --- | --- | --- | --- | --- | --- | --- | --- | --- | --- | --- | --- | --- |
|  | **Total** | **Expected** | **Hits** | **Raw_p** | **Holm_p** | **FDR** | **Total** | **Expected** | **Hits** | **Raw_p** | **Holm_p** | **FDR** | **Total** | **Expected** | **Hits** | **Raw_p** | **Holm_p** | **FDR** |
| **Phenylalanine, tyrosine and tryptophan biosynthesis** | 4 | 0.05 | 2 | 0 | 0.08 | 0.07 | 4 | 0.02 | 1 | 0.02 | 1 | 0.61 | 4 | 0.02 | 1 | 0.02 | 1 | 0.38 |
| **Nitrogen metabolism** | 6 | 0.08 | 2 | 0 | 0.2 | 0.07 | 6 | 0.03 | 1 | 0.03 | 1 | 0.61 | 6 | 0.03 | 1 | 0.03 | 1 | 0.43 |
| **Phenylalanine metabolism** | 8 | 0.1 | 2 | 0 | 0.36 | 0.09 | 8 | 0.05 | 1 | 0.05 | 1 | 0.67 | 8 | 0.05 | 1 | 0.05 | 1 | 0.5 |
| **Glutathione metabolism** | 28 | 0.38 | 2 | 0.05 | 1 | 0.53 | 28 | 0.18 | 2 | 0.01 | 1 | 0.52 | 28 | 0.18 | 2 | 0.01 | 1 | 0.26 |
| **Tryptophan metabolism** | 41 | 0.55 | 4 | 0 | 0.15 | 0.07 | 41 | 0.26 | 4 | 0 | 0.01 | 0.01 |  |  |  |  |  |  |
| **Alanine, aspartate and glutamate metabolism** | 28 | 0.38 | 3 | 0.01 | 0.44 | 0.09 |  |  |  |  |  |  | 28 | 0.18 | 2 | 0.01 | 1 | 0.26 |
| **Arginine biosynthesis** | 14 | 0.19 | 2 | 0.01 | 1 | 0.2 |  |  |  |  |  |  | 14 | 0.09 | 2 | 0.01 | 0.265 | 0.17 |
| **Linoleic acid metabolism** |  |  |  |  |  |  | 5 | 0.03 | 1 | 0.03 | 1 | 0.61 |  |  |  |  |  |  |
| **Histidine metabolism** |  |  |  |  |  |  |  |  |  |  |  |  | 16 | 0.1 | 2 | 0.01 | 0.343 | 0.17 |
| **Tyrosine metabolism** |  |  |  |  |  |  |  |  |  |  |  |  | 42 | 0.27 | 2 | 0.03 | 1 | 0.38 |
